# Information Retrieval in an Infodemic: The Case of COVID-19 Publications

**DOI:** 10.2196/30161

**Published:** 2021-09-17

**Authors:** Douglas Teodoro, Sohrab Ferdowsi, Nikolay Borissov, Elham Kashani, David Vicente Alvarez, Jenny Copara, Racha Gouareb, Nona Naderi, Poorya Amini

**Affiliations:** 1 Geneva School of Business Administration HES-SO University of Applied Arts and Sciences of Western Switzerland Carouge Switzerland; 2 Swiss Institute of Bioinformatics Lausanne Switzerland; 3 Department of Radiology and Medical Informatics University of Geneva Geneva Switzerland; 4 Risklick AG Bern Switzerland; 5 Clinical Trials Unit Bern Switzerland; 6 Institute of Pathology University of Bern Bern Switzerland

**Keywords:** information retrieval, multistage retrieval, neural search, deep learning, COVID-19, coronavirus, infodemic, infodemiology, literature, online information

## Abstract

**Background:**

The COVID-19 global health crisis has led to an exponential surge in published scientific literature. In an attempt to tackle the pandemic, extremely large COVID-19–related corpora are being created, sometimes with inaccurate information, which is no longer at scale of human analyses.

**Objective:**

In the context of searching for scientific evidence in the deluge of COVID-19–related literature, we present an information retrieval methodology for effective identification of relevant sources to answer biomedical queries posed using natural language.

**Methods:**

Our multistage retrieval methodology combines probabilistic weighting models and reranking algorithms based on deep neural architectures to boost the ranking of relevant documents. Similarity of COVID-19 queries is compared to documents, and a series of postprocessing methods is applied to the initial ranking list to improve the match between the query and the biomedical information source and boost the position of relevant documents.

**Results:**

The methodology was evaluated in the context of the TREC-COVID challenge, achieving competitive results with the top-ranking teams participating in the competition. Particularly, the combination of bag-of-words and deep neural language models significantly outperformed an Okapi Best Match 25–based baseline, retrieving on average, 83% of relevant documents in the top 20.

**Conclusions:**

These results indicate that multistage retrieval supported by deep learning could enhance identification of literature for COVID-19–related questions posed using natural language.

## Introduction

### Background

In parallel with its public health crisis with vast social and economic impacts, the COVID-19 pandemic has resulted in an explosive surge of activities within scientific communities and across many disciplines [1]. In turn, it has led to an overabundance of information online and offline — a phenomenon described as an infodemic [2-4] — with often negative impacts on the population [5]. Since early 2020 when the pandemic was officially announced, the number of publications related to COVID-19 has had exponential growth [6]. In addition to the volume and velocity of the generated data, the heterogeneity as a result of the typical variety of concept naming found in the biomedical field, spelling mistakes, and the different source types [7] make searching and discovery of relevant literature within the COVID-19 corpora an important challenge [2].

With the sheer quantity of COVID-19 information continuously produced, researchers, policy makers, journalists, and ordinary citizens, among others, are unable to keep up with the fast-evolving body of knowledge disseminated. As knowledge about the pandemic evolves, study results and conclusions may be improved, contradicted, or even proven wrong [3]. Combined with relentless media coverage and social media interactions, this fast-changing and massive amount of information leads to confusion and desensitization among audiences (eg, as in the case of school opening guidelines and mask-wearing and social distancing recommendations) [5,8]. They also fuel deliberate attempts to create information disorders, such as misinformation, disinformation, malinformation, and fake news [9], reducing the effectiveness of public health measures and endangering countries’ ability to stop the pandemic, ultimately having a negative impact on live costs [10,11].

To support states and relevant actors of society to manage the COVID-19 infodemic, the World Health Organization (WHO) has published a framework containing 50 recommendations, of which more than 20% are related to strengthening the scanning, review, and verification of evidence and information [2]. To help actors involved with the pandemic find the most relevant information for their needs, effective information retrieval models for the COVID-19–related corpora became thus a prominent necessity [12]. The information retrieval community, in turn, has responded actively and quickly to this extraordinary situation and has been aiming to address these challenges. To foster research for the scientific communities involved with the pandemic, the COVID-19 Open Research Dataset (CORD-19) [13] collection was built to maintain all the related publications for the family of coronaviruses. This dataset helped research in various directions, and several tasks are built around it, including natural language processing (NLP)–related tasks, like question answering [14] and language model pretraining [15], and information retrieval challenges in Kaggle [16] as well as the TREC-COVID [17,18].

The TREC-COVID [18-20] challenge ran in 5 rounds, each asking for an incremental set of information needs to be retrieved from publications of the CORD-19 collection. In a TREC-COVID round, participants were asked to rank documents of the CORD-19 corpus in decreasing order of likelihood of containing answers to a set of query topics. At the end of the round, experts provided relevance judgments for the top-ranking documents submitted by different participants using a pooling strategy [21]. Although limited to the first several top submissions of the participating teams, these relevance judgments enable the evaluation of the different models and are valuable examples to train retrieval models for the subsequent rounds of the challenge.

To improve search and discovery of COVID-19 scientific literature, in this work we aimed to investigate an information retrieval model supported by deep language models to enhance findability of relevant documents in fast-evolving corpora.

More than 50 teams participated in the TREC-COVID challenge worldwide, developing new information retrieval and NLP methodologies to tackle this complex task [22-27]. Having participated in the TREC-COVID challenge, in this paper we detail our retrieval methodology, which brought us competitive results with the top-ranking teams. Particularly, we used a multistage retrieval pipeline, combining classic probabilistic weighting models with novel learning to rank approaches made by ensemble of deep masked language models. We present our results and analyze how the different components of the pipeline contribute to providing the best answers to the query topics.

### Related Work

#### Two-Stage Information Retrieval

Currently, 2 main methodologies are used to rank documents in information retrieval systems: (1) the classic query-document probabilistic approaches, such as Okapi Best Match 25 (BM25) [28] and probabilistic language models [29], and (2) the learning-to-rank approaches, which usually postprocess results provided by classic systems to improve the original ranked list [30,31]. When there are sufficient training data (ie, queries with relevance judgments for the case of information retrieval), learning-to-rank models often outperform classic one-stage retrieval systems [30,32]. Nevertheless, empiric results have also shown that the reranking step may degrade the performance of the original rank [33]. Progress on learning-to-rank algorithms has been fostered thanks to the public release of annotated benchmark datasets, such as the LETOR [34] and Microsoft Machine Reading Comprehension (MS MARCO) [35].

Learning-to-rank approaches can be categorized into 3 main classes of algorithms — pointwise, pairwise, and listwise — based on whether they consider 1 document, a pair of documents, or the whole ranking list in the learning loss function, respectively [30-32,36]. Variations of these learning-to-rank algorithms are available based on neural networks [31,36] and other learning algorithms, such as boosting trees [37]. More recently, pointwise methods leveraging the power of neural-based masked language models have attracted great attention [38,39]. These learning-to-rank models use the query and document learning representations provided by the masked language model to classify whether a document in the ranked list is relevant to the query. While these two-stage retrieval methods based on neural rerankers provide interesting features, such as learned word proximity, in practice, the first stage based on classic probabilistic retrieval algorithms is indispensable, as the algorithmic complexity of the reranking methods makes them often prohibitive to classify the whole collection [32].

Recent advances in text analytics, including question answering, text classification, and information retrieval, have indeed mostly been driven by neural-based masked language models. A seminal effort in this direction is the Bidirectional Encoder Representations from Transformers (BERT) model [38], which shows significant success in a wide range of NLP tasks. BERT uses a bidirectional learning approach based on the transformer architecture [40] and is trained to predict masked words in context. Since the introduction of BERT, several works tried to augment its performance. A successful work in this direction is the robustly optimized BERT approach (RoBERTa) [41], using larger and more diverse corpora for training as well as a different tokenizer. While RoBERTa needs larger computing power, it often improves the performance of BERT across different downstream tasks. Another similar effort is the XLNet model [42], which uses a permutation-based masking, showing also consistent improvement over BERT.

#### TREC-COVID Retrieval Efforts

Recently, the specific case of retrieval of COVID-related scientific publications has been addressed in several efforts [22-27]. These works follow mostly the aforementioned two-stage retrieval process. Among the first efforts is the SLEDGE system [22], where the authors detailed their solutions for the first round of the TREC-COVID challenge using a BM25-based ranking method followed by a neural reranker. An important difficulty for the first round of the challenge is the absence of labelled data. To overcome this limitation, the authors lightly tuned the hyperparameters of the first-stage ranking model using minimal human judgments on a subset of the topics. As for the second stage, they used the SciBERT model [43], which is pretrained on biomedical texts, and fine-tuned on the general MS MARCO set [35] with a simple cross-entropy loss. CO-Search [24] uses a slightly different approach, wherein they incorporated semantic information, as captured by Sentence-BERT [44], also within the initial retrieval stage. Moreover, they used the citation information of publications in their ranking pipeline. In the work of Covidex [23], the authors provided a full-stack search engine implementing a multistage ranking pipeline, where their first stage is based on the Anserini information retrieval toolkit [45], complemented by different neural reranking strategies. They addressed the issue of length variability among documents with an atomic document representation using, for example, paragraph-level indexing.

## Methods

In this section, we describe the corpus and query set and our methodology for searching COVID-19–related literature in the context of the TREC-COVID challenge. We start by introducing the CORD-19 dataset, which is the corpus used in the competition. We then describe the challenge organization and assessment queries. Then, we detail our searching methodology, based on a multistage retrieval approach. Finally, we present the evaluation criteria used to score the participants’ submissions. For further details on the TREC-COVID challenge, see [19,20].

### The CORD-19 Dataset

**Figure 1:**
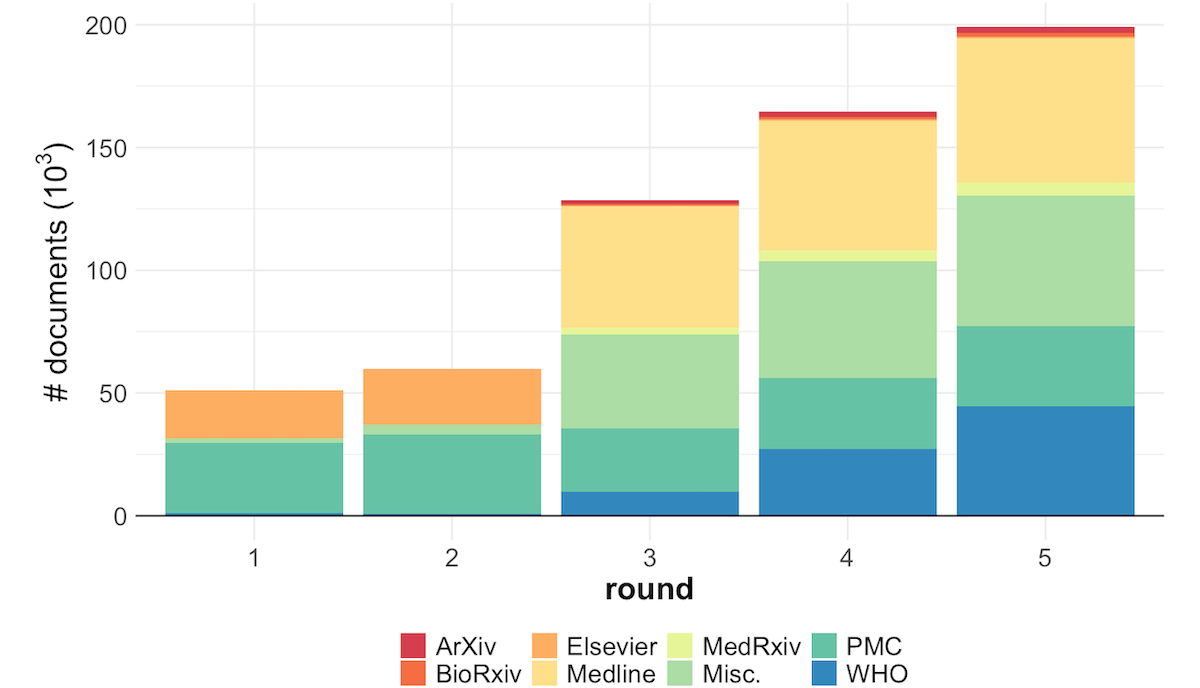
Evolution of the CORD-19 corpus across the TREC-COVID rounds stratified by source. PMC: PubMed Central; WHO: World Health Organization.

A prominent effort to gather publications, preprints, and reports related to the coronaviruses and acute respiratory syndromes (COVID-19, Middle East respiratory syndrome [MERS], and severe acute respiratory syndrome [SARS]) is the CORD-19 collection of the Allen Institute for Artificial Intelligence (in collaboration with other partners) [13]. Figure 1 describes the size and content origin of the corpus for the different TREC-COVID rounds. As we can see, this is a large and dynamically growing semistructured dataset from various sources like PubMed, PubMed Central (PMC), WHO, and preprint servers like bioRxiv, medRxiv, and arXiv. The dataset contains document metadata, including title, abstract, and authors, among others, but also the full text or link to full-text files when available. A diverse set of related disciplines (eg, from virology and immunology to genetics) are represented in the collection. Throughout the challenge, the dataset was updated daily, and snapshot versions representing its status at a certain time were provided to the participants for each round. In the last round of the TREC-COVID challenge, the corpus contained around 200,000 documents, coming mostly from Medline, PMC, and WHO sources.

### The TREC-COVID Challenge

To assess the different information retrieval models, the TREC-COVID challenge provided a query set capturing relevant search questions of researchers during the pandemic. These needs are stated in query topics, consisting of 3 free-text fields — query, question, and narrative — with an increasing level of context, as shown in the examples in Table 1. The challenge started with 30 topics in round 1 and added 5 new topics in each new round, thus reaching 50 topics in round 5.

In each round, the participants provided ranked lists of candidate publications of the CORD-19 collection that best answered the query topics. Each list was generated by a different information retrieval model, so called *run*, with up to 5 runs in the first 4 rounds and 7 runs in the last round per team. At the end of the round, domain experts examined the top k candidate publications (where k is defined by the organizers) from the priority runs of the teams and judged them as “highly relevant,” “somehow relevant,” or “irrelevant.” Then, based on the consolidated relevance judgments, the participants were evaluated using standard information retrieval metrics (eg, normalized discounted cumulative gain [NDCG], precision). Judged documents for a specific topic from previous rounds were excluded from the relevance judgment list.

**Table 1:** Examples of TREC-COVID topics with the fields query, question, and narrative.

Topic	Query	Question	Narrative
1	Coronavirus origin	What is the origin of COVID-19?	Seeking a range of information about the SARS-CoV-2 virus’s origin, including its evolution, animal source, and first transmission into humans
25	Coronavirus biomarkers	Which biomarkers predict the severe clinical course of 2019-nCOV infection?	Looking for information on biomarkers that predict disease outcomes in people infected with coronavirus, specifically those that predict severe and fatal outcomes
50	mRNA vaccine coronavirus	What is known about an mRNA vaccine for the SARS-CoV-2 virus?	Looking for studies specifically focusing on mRNA vaccines for COVID-19, including how mRNA vaccines work, why they are promising, and any results from actual clinical studies

### Proposed Multistage Retrieval Methodology

**Figure 2:**
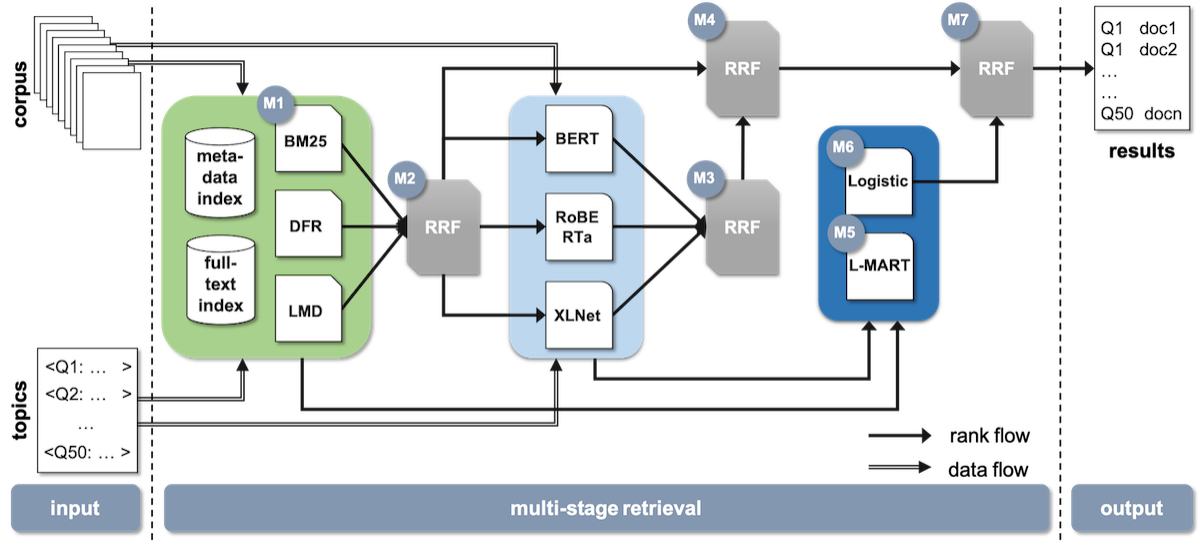
Multistage retrieval pipeline, where light green indicates first-stage retrieval, light and dark blue indicate second-stage retrieval, and M1-M7 denote the different models used to create the respective runs 1-7 in round 5. BERT: Bidirectional Encoder Representations from Transformers; BM25: Okapi Best Match 25; DFR: divergence from randomness; L-MART: LambdaMART model; LMD: language model Dirichlet; Logistic: logistic regression model; RoBERTa: robustly optimized BERT approach; RRF: reciprocal rank fusion.

Figure 2 shows the different components of our information retrieval pipeline for the COVID-related literature. These components can be divided into 3 main categories: (1) first-stage retrieval using classic probabilistic methods, (2) second-stage (neural) reranking models, and (3) rank fusion algorithms. Given a corpus containing metadata information, such as title and abstract, and full text, when available, documents are stored using directed and inverted indexes. Then, transformer-based and classic learning-to-rank models trained using relevance judgments are used to classify and rerank pairs of query-document answers. The ranked list obtained from the different models are further combined using the reciprocal rank fusion (RRF) algorithm.

#### First-Stage Retrieval

For the first-stage retrieval, we assessed 3 variations of the classic query-document probabilistic weighting models: BM25 [46], divergence from randomness (DFR) [47], and language model Dirichlet (LMD) [48].

Our first classical model, Okapi BM25 [28], is based on the popular term frequency-inverse document frequency (tf-idf) framework. In the tf-idf framework, term-weights are calculated using the product of within term-frequency *tf* and the inverse document frequency *idf* statistics. Denote *f(t,d)* as the number of times a term *t* appears in a document *d* within a collection *D*, BM25 calculates the term-weight *w* as:




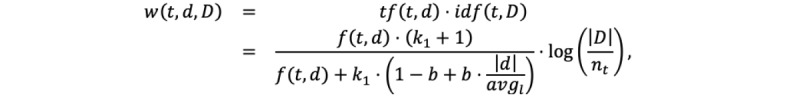




where |*d*| is the length of the document, |*D*| is the size of the collection, *avg_l_* is the average length of the documents in the collection, *n_t_* is the number of documents containing the term *t*, and *k*_1_ and *b* are parameters of the model associated with the term frequency and the document size normalization, respectively.

The second model, DFR, extends the basic tf-idf concept by considering that the more the divergence of the term frequency *tf* from its collection frequency *cf* (*cf ≈ df*), the more the information carried by the term in the document [47]. Thus, for a given model of randomness *M*, in the DFR framework, the term-weight is inversely proportional to the probability of term-frequency within the document obtained by *M* for the collection *D*:

w(t,d,D) = k·logp_M_ (t ϵ d|D),

where *p_M_* is a probabilistic model, such as binomial or geometric distributions, and *k* is a parameter of the probabilistic model.

The third model, LMD, uses a language model that assigns probabilities to word sequences with a Dirichlet-prior smoothing to measure the similarity between a query and a document [48]. In a retrieval context, a language model specifies the probability that a document is generated by a query, and smoothing is used to avoid zero probabilities to unseen words and improves the overall word probability accuracy. In the LMD algorithm, term-weight is calculated using the following equation:




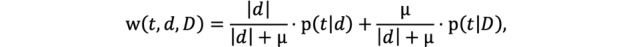




where *p*(*t*|*d*) denotes the probability of a term in a document, *p*(*t*|*D*) is the probability of a term in the collection, and *μ* is the Dirichlet parameter to control the amount of smoothing.

In our pipeline, the BM25, DFR, and LMD implementations are based on the Elasticsearch framework. The model parameters were trained using the queries and relevance judgments of round 4 in a 5-fold cross-validation setup.

#### Second-Stage Reranking

The models used in the first-stage ranking were based on the bag-of-words statistics, where essentially we looked at the histogram of query terms and their document and collection statistics but neglected the sequential nature of text and word relations. To mitigate these limitations and improve the initial rankings, after the first-stage retrieval, we used neural masked language models trained on the relevance judgments from previous rounds so that syntactic and semantic relations can be better captured [49,50]. As shown in Figure 2, we assessed 3 masked language models based on the transformer architecture: BERT, RoBERTa, and XLNet.

**Figure 3:**
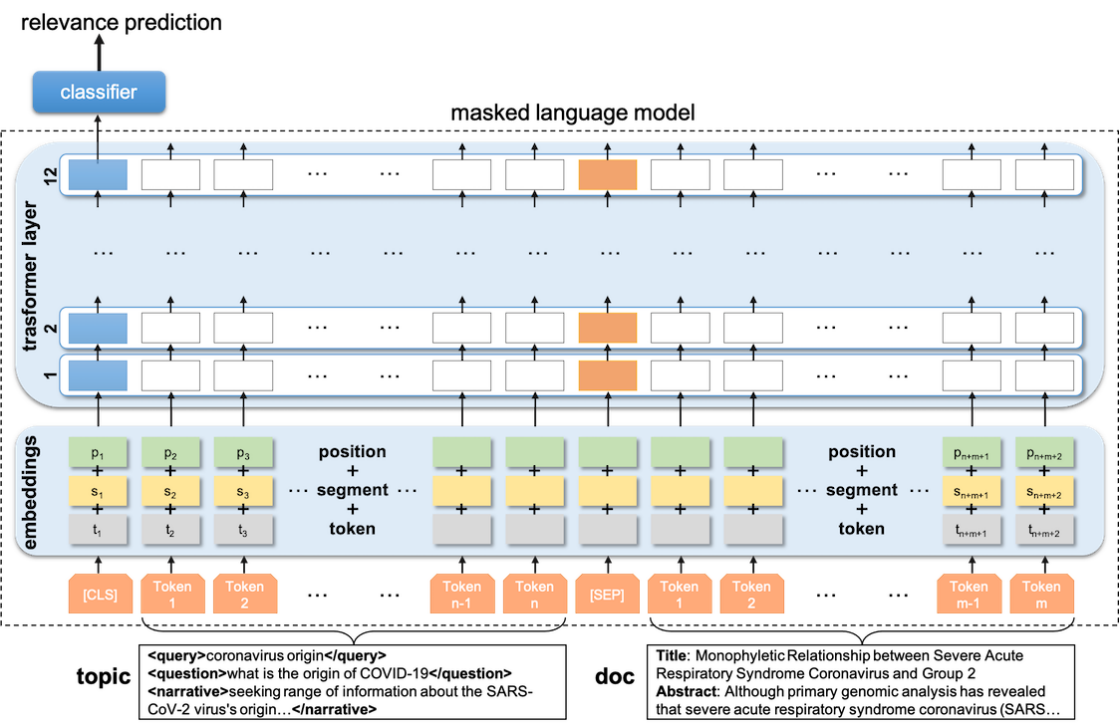
Neural masked language model for document relevance classification. As inputs to the pre-trained masked language model, the topics and candidate publications are separated by the [SEP] tag. Inputs are tokenized using subword tokenization methods (grey boxes); segment embeddings (yellow boxes) represent the difference between a topic and a document input; position embeddings (green boxes) enforce the sequential structure of text; the transformer and classification layers are updated in the training phase using the relevance judgments; and the output of the special [CLS] token is finally used to determine the relevance of the candidate publication to the queried information topic.

Figure 3 shows the general idea of how we used the BERT language model to match documents to a query topic. Given a topic and a document associated with it as input and a relevance judgment as the label for the query-document association (relevant or not), the model was trained or fine-tuned in the BERTology parlance, as it had been previously pretrained on a large corpus, to predict whether the document is relevant to the query. In the input layer of the pretrained model, the topic and candidate publication were tokenized and separated by the language model *[SEP]* token (stands for sentence separation). Moreover, to enforce the sequential structure of text, positional embedding as well as sentence embeddings were added to the main embeddings for each token. These embeddings were then fed to the transformer layers of BERT, which were updated during the fine-tuning step. Finally, the output of the special *[CLS]* token (stands for classification) was used to determine the relevance of the candidate publication to the queried information topic.

Using the query topics from a preceding round (round 4 for the results presented here) and their respective list of relevance judgments, we fine-tuned the BERT model to rescore the initial association of the query-document pair between (not relevant) and (very relevant). For this, we used the score associated with the *[CLS]* token position. We limited the input size of the query and document to 512 tokens (or subwords). Then, at the second-stage re-ranking step, we classified the top *k* publications retrieved by the first stage models using the fine-tuned BERT model (we set *k*=5000 in our experiments).

Identical training strategies were used for the RoBERTa and XLNet language models. The main difference for the RoBERTa model is that it was originally pretrained on a corpus with an order of magnitude bigger than that of BERT (160 GB vs 16 GB). Moreover, it uses dynamic masking during the training process, that is, at each training epoch, the model sees different versions of the same sentence with masks on different positions, compared to a static mask algorithm for BERT. Last, RoBERTa uses a byte-level Byte-Pair-Encoding tokenizer compared to BERT’s WordPiece. As BERT and its variants (eg, RoBERTa) neglect the dependency between the masked positions and suffer from a pretrain-finetune discrepancy, XLNet adopts a permutation language model instead of masked language model to solve the discrepancy problem. For downstream tasks, the fine-tuning procedure of XLNet is similar to that of BERT and RoBERTa.

We used the BERT (base - 12 layers), RoBERTa, and XLNet model implementations available from the Hugging Face framework. The models were trained using the Adam optimizer [51] with an initial learning rate of 1.5e–5, weight decay of 0.01, and early stopping with a patience of 5 epochs.

#### Combining Model Results

We used the RRF algorithm [52] to combine the results of different retrieval runs. RRF is a simple, yet powerful technique to rescore a retrieval list based on the scores of multiple retrieval lists. Given a set of documents *D* to be sorted and a set of ranking files *R* = {*r*_1_…*r*_n_}, each with a permutation on 1…|*D*|, RRF computes the aggregated score using the following equation:




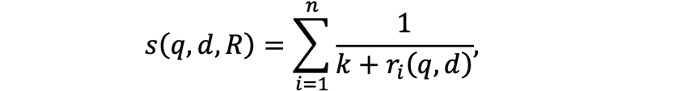




where *r*(*q,d*) is the rank of document *d* for the query *q* in the ranking file *r_i_* and *k* is a threshold parameter, which was tuned to *k*=60 using data from previous rounds.

#### Second-Step Learning-to-Rank

To exploit the features (relevance score) created by the different bag-of-words and masked language models, we added a second-step learning-to-rank pass to our pipeline. Using the similarity scores *s* computed by the BM25, DFR, LMD, BERT, RoBERTa, and XLNet as input features and the relevance judgments of previous rounds as labels, we trained 2 learning-to-rank models: LambdaMART and a logistic regression classifier. While the language models exploit the sequential nature of text, they completely neglect the ranking provided by the bag-of-words models. Thus, we investigated the use of the LambdaMART [31] algorithm, which uses a pairwise loss that compares pairs of documents and tells which document is better in the given pair. Moreover, we trained a simple pointwise logistic regression to consider the similarity measures computed by the first- and second-stage retrieval models. We used the pyltr and scikit-learn implementations for the LambdaMART and logistic regression, respectively. For the LambdaMART model, we trained the learning rate and the number of estimators, and for the logistic regression model, we trained the solver and regularization strength parameters.

#### First-Stage Retrieval: Preprocessing, Querying Strategies, and Parameter Tuning

In the first-stage retrieval step, we applied a classical NLP preprocessing pipeline to the publications (indexing phase) and topics (search phase): lower-casing, removal of nonalphanumerical characters (apart from “-”), and Porter stemming. Additionally, a minimal set of COVID-related synonyms, such as “covid-19” and “sars-cov-2,” were created and used for query expansion.

The queries were then submitted to the index in a combinatorial way using the different topic fields and document sections. This means that, for each *query*, *question*, and *narrative* field of a topic, we submitted a query against the index for each of the *title* and *abstract* sections of the publications (abstract + full text in case of the full-text index). Additionally, the whole topic (query + question + narrative) was queried against the whole document. This querying strategy led to 7 queries for each topic, and the final score was computed by summing up the individual scores. Moreover, as the first public announcement of a coronavirus-related pneumonia was made in January 2020, we filtered out all publications before December 2019.

We defined the best query strategy and fine-tuned the basic parameters of the bag-of-words models using the relevance judgments of the previous round in a 5-fold cross-validation approach. As an example, to tune the *b* and *k* parameters of the BM25 model in round 5, we took the topics and relevance judgment from round 4 and submitted them to the index in round 5, optimizing the P@10 metric. For round 1, we used default parameter values.

### Evaluation Criteria

We used the official metrics of the TREC-COVID challenge to report our results: precision at K documents (P@K), NDCG at K documents (NDCG@K), mean average precision (MAP), and binary preference (Bpref) [19]. For all these metrics, the closest the result is to 1, the better the retrieval model. They were obtained using the *trec_eval* information retrieval evaluation toolkit.

## Results

We used 7 models from our pipeline to create the 7 runs submitted for the official evaluation of the TREC-COVID challenge (labels M1 to M7 in Figure 2). Our first model — *bm25* — based on the BM25 weighting model against the metadata index provided the baseline run. Our second model — *bow + rrf* — was a fusion of the BM25, DFR, and LMD weighting models computed against the metadata and full-text indices and combined using the RRF algorithm. Model 3 — *mlm + rrf* — used the RRF combination of the BERT, RoBERTa, and XLNet models applied to the top 5000 documents retrieved by model 2. Model 4 — *bow + mlm + rrf* — combined the results of models 2 and 3 using the RRF algorithm. Then, model 5 — *bow + mlm + lm* — reranked the results of runs 2 and 3 using the LambdaMART algorithm trained using the similarity scores of the individual models 2 and 3. Similarly, model 6 — *bow + mlm + lr* — was based on a logistic regression classifier that uses as features the similarity scores of runs 2 and 3 to classify the relevance of the query-document pairs. Finally, model 7 — *bow + mlm + lr + rrf* — combined runs 2, 3, and 6 using the RRF algorithm. For all RRF combinations, the parameter *k* was set to 60. All models and parameters were trained using round 4 relevance judgments. Table 2 summarizes the submitted runs.

**Table 2:** Summary of the submitted runs. Refer to Figure 2 for a pictorial description.

Run	Name	Description
1	bm25	Run based on the baseline BM25^a^ model using the metadata index
2	bow + rrf	An RRF^b^ combination of BM25, DFR^c^, and LMD^d^ models computed against the metadata and full-text indices
3	mlm + rrf	An RRF combination of BERT, RoBERTa^e^, and XLNet models applied to run 2
4	bow + mlm + rrf	An RRF combination of runs 2 and 3
5	bow + mlm + lm	A LambdaMART-based model using features from the individual models used to create runs 2 and 3
6	bow + mlm + lr	A logistic regression model using features from the individual models used to create runs 2 and 3
7	bow + mlm + lr + rrf	An RRF combination of runs 2, 3, and 6

^a^BM25: Okapi Best Match 25.

^b^RRF: reciprocal rank fusion.

^c^DFR: divergence from randomness.

^d^LMD: language model Dirichlet.

^e^RoBERTa: robustly optimized BERT approach.

### Official Evaluation Results

Table 3 shows the official results of the TREC-COVID challenge for the 7 submitted runs. As we can see, the best results are provided by model 7 (bow + mlm + lr + rrf), apart from the metric Bpref, which is the highest for model 5 (bow + mlm + lm). Comparing the NDCG@20 metric, model 7 improved 16.4 percentage points against the baseline model (26.0% relative improvement). On average, almost 17 of the top 20 documents retrieved by model 7 were pertinent to the query. Model 3 was able to retrieve 6.6% more relevant documents compared to the baseline model (6963 vs 6533 of a total of 10,910 documents judged relevant for the 50 queries). On the other hand, it showed a relative improvement in precision of 22.1% for the top 20 documents. Therefore, it not only improved the recall but also brought relevant documents higher in the ranking list. These results show that the use of the masked language models had a significant positive impact in the ranking.

**Table 3:** Performance of our models in round 5 of the TREC-COVID challenge.

Model	NDCG@20^a^	P@20^b^	Bpref^c^	MAP^d^	# rel^e^
bm25	0.6320	0.6440	0.5021	0.2707	6533
bow + rrf	0.6475	0.6650	0.5174	0.2778	6695
mlm + rrf	0.7716	0.7880	0.5680	0.3468	6963
bow + mlm + rrf	0.7826	0.8050	0.5616	0.3719	7006
bow + mlm + lm	0.7297	0.7460	0.5759	0.3068	6834
bow + mlm + lr	0.7375	0.7450	0.5719	0.3439	6976
bow + mlm + lr + rrf	0.7961	0.8260	0.5659	0.3789	6939

^a^NDCG@20: normalized discounted cumulative gain at 20 documents.

^b^P@20: precision at 20 documents.

^c^Bpref: binary preference.

^d^MAP: mean average precision.

^e^# ref: total number of relevant documents retrieved by the model for the 50 queries.

Table 4 shows the official best results for the different metrics for the top 10 teams participating in round 5 of TREC-COVID (NDCG@20 metric taken as reference). Comparing the NDCG@20 metric, the best model submitted by our team (risklick) was ranked 4 of the 28 teams participating in round 5, 5.4 percentage points below the top-performing team (Unique-ptr). For reference, the best-performing model in the challenge retrieves on average 17.5 relevant documents per query in the top 20 retrieved documents compared to 16.5 for our model. If we consider a reference baseline made by the median of the participating teams’ best values, our pipeline outperforms the baseline by 11.7%, 14.6%, 16.7%, and 25.0% for the MAP, P@20, NDCG@20, and Bpref metrics, respectively. All data and results of the TREC-COVID challenge can be found here: [17].

**Table 4:** Official leaderboard of the top 10 teams in the final round of the TREC-COVID challenge.

Team	NDCG@20^a^	P@20^b^	Bpref^c^	MAP^d^
unique_ptr	0.8496	0.8760	0.6378	0.4731
covidex	0.8311	0.8460	0.5330	0.3922
Elhuyar_NLP_team	0.8100	0.8340	0.6284	0.4169
risklick (ours)	0.7961	0.8260	0.5759	0.3789
udel_fang	0.7930	0.8270	0.5555	0.3682
CIR	0.7921	0.8320	0.5735	0.3983
uogTr	0.7921	0.8420	0.5709	0.3901
UCD_CS	0.7859	0.8440	0.4488	0.3348
sabir	0.7789	0.8210	0.6078	0.4061
mpiid5	0.7759	0.8110	0.5873	0.3903

^a^NDCG@20: normalized discounted cumulative gain at 20 documents.

^b^P@20: precision at 20 documents.

^c^Bpref: binary preference.

^d^MAP: mean average precision.

### Model Performance Analyses

**Figure 4:**
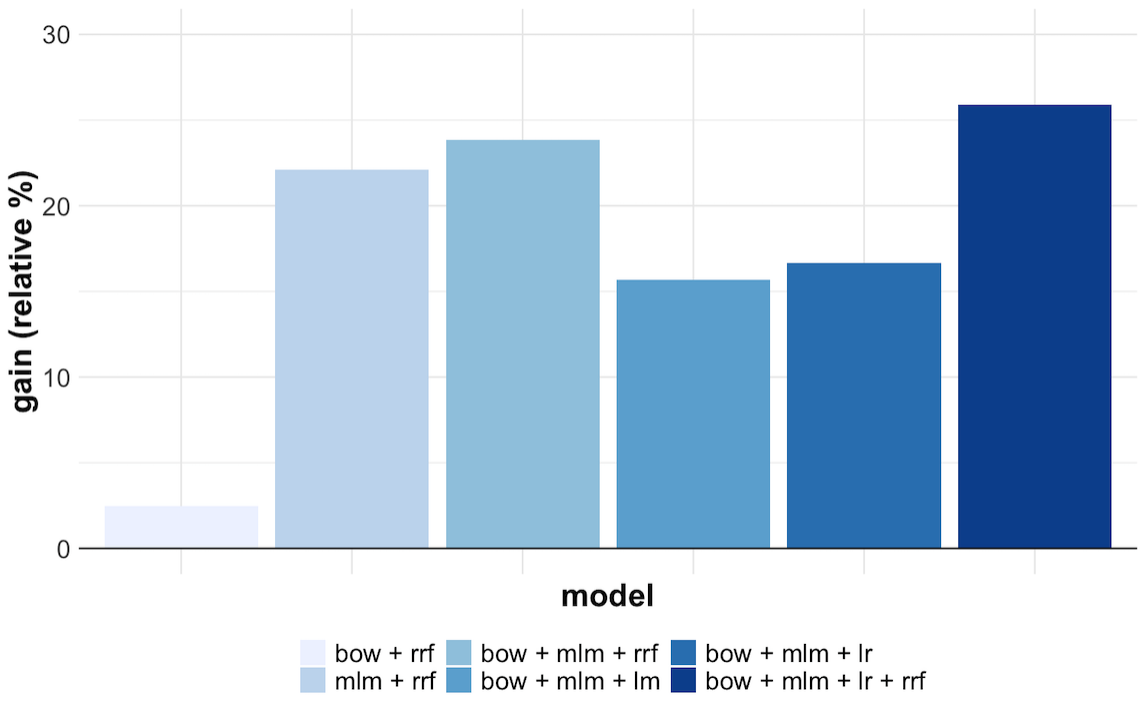
Relative contribution of each model for the normalized discounted cumulative gain at document 20 (NDCG@20) metric compared to the baseline model bm25.

Figure 4 shows the relative improvement of the different models in the pipeline in relation to the baseline (model 1 – bm25) according to the NDCG@20 metric. The most significant contribution to the final performance came from the inclusion of the masked language models in the pipeline — model 3: mlm + rrf and model 4: bow + mlm + rrf — adding a relative performance gain to the results of 22.1% and 23.8%, respectively. The classic learning-to-rank models — model 5 and model 6 — actually jeopardized the performance when compared to their previous model in the pipeline (model 4). However, when model 6 was combined with model 4, a 2.1 percentage point gain was achieved on top of model 4, leading to the best model (model 7: bow + mlm + lr + rrf). Indeed, it is important to notice the consistent benefit of combining models using the RRF algorithm. Interestingly, the effect of LambdaMART seemed to be significantly detrimental for P@20, NDCG@20, and MAP, but marginally beneficial for Bpref, for which it is the best model.

The performance of the individual masked language models is shown in Table 5. Surprisingly, they are similar to the baseline model, with small performance reductions for BERT and RoBERTa models and a small performance gain for the XLNet model. However, when combined, they provide the significant performance improvement shown in Figure 4. Our assumption is that they retrieve different documents as relevant and their combination using RRF ends up aligning these documents in the top rank. Indeed, looking at the top 3 documents for query 1 retrieved by these models, for example, there is no overlap between the documents, with 8 relevant and 1 unjudged (out of the 9 documents). This result clearly shows the beneficial effect of using an ensemble of masked language models, as well as the success of RRF in fusing their retrievals.

**Table 5:** Performance of the individual masked language models and their combination using reciprocal rank fusion (RRF).

Model	NDCG@20^a^	P@20^b^	Bpref^c^	MAP^d^	# rel^e^
BERT^f^	0.6209	0.6430	0.5588	0.2897	6879
RoBERTa^g^	0.6261	0.6440	0.5530	0.2946	6945
XLNet	0.6436	0.6570	0.5644	0.3064	6926
mlm + rrf	0.7716	0.7880	0.5680	0.3468	6963

^a^NDCG@20: normalized discounted cumulative gain at 20 documents.

^b^P@20: precision at 20 documents.

^c^Bpref: binary preference.

^d^MAP: mean average precision.

^e^# ref: total number of relevant documents retrieved by the model for the 50 queries.

^f^BERT: Bidirectional Encoder Representations from Transformers.

^g^RoBERTa: robustly optimized BERT approach.

### Topic Performance Analyses

**Figure 5:**
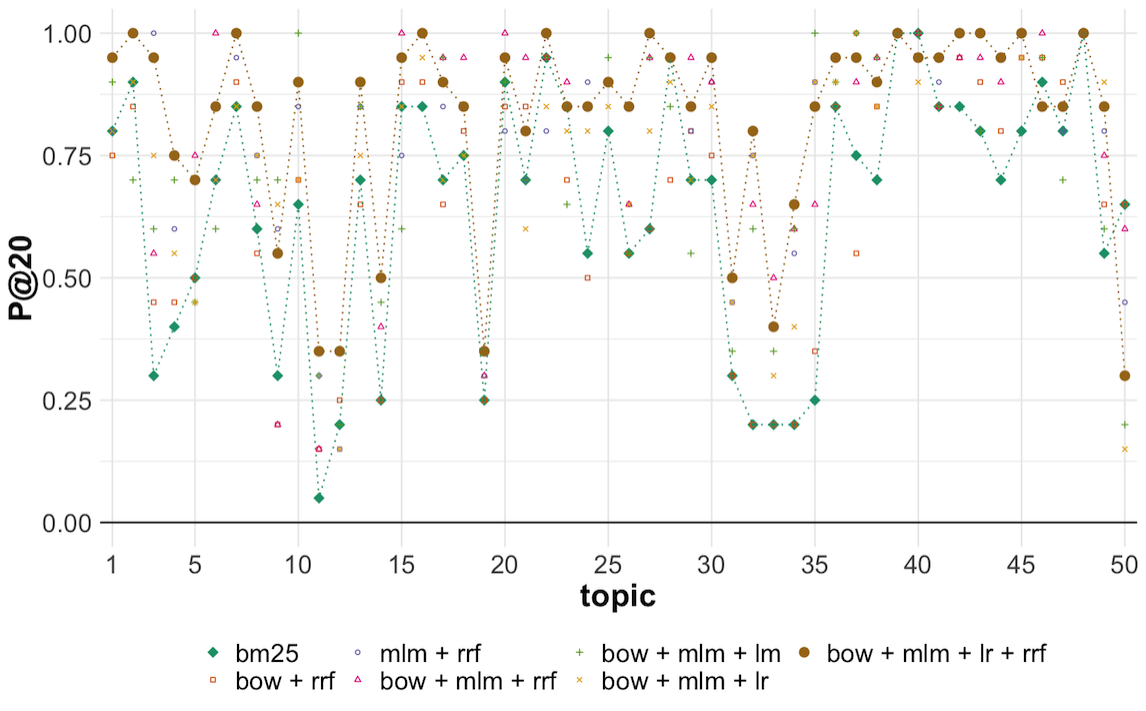
Per topic precision at rank 20 (P@20) in round 5 of TREC-COVID per each run. The baseline run1 and the best-performing run7, which benefits from neural language models, are highlighted with dashed lines. Note that for most topics, the transformer-based runs have significantly improved performance.

The performance analyses for the individual topics shows that our best model had a median value of 0.9000 for the P@20 metric (max=1.0000, min=0.3000), which demonstrates successful overall performance. However, as shown in Figure 5, for some topics, notably 11, 12, 19, 33, and 50, less than 50% of documents in the top 20 retrieved are relevant. For topics 11, 12, and 19, which searched for “coronavirus hospital rationing,” “coronavirus quarantine,” and “what alcohol sanitizer kills coronavirus” information, respectively, all our models have poor performance, and indeed, the combination of the different models in the pipeline managed to boost the results. On the other hand, for topics 33 and 50, which searched for “coronavirus vaccine candidates” and “mRNA vaccine coronavirus” information, respectively, it was the combination with the logistic regression model that lowered the performance (notice in Figure 5 that model 4: bow + mlm + rrf has a significantly better performance compared to model 7 for those queries).

**Figure 6:**
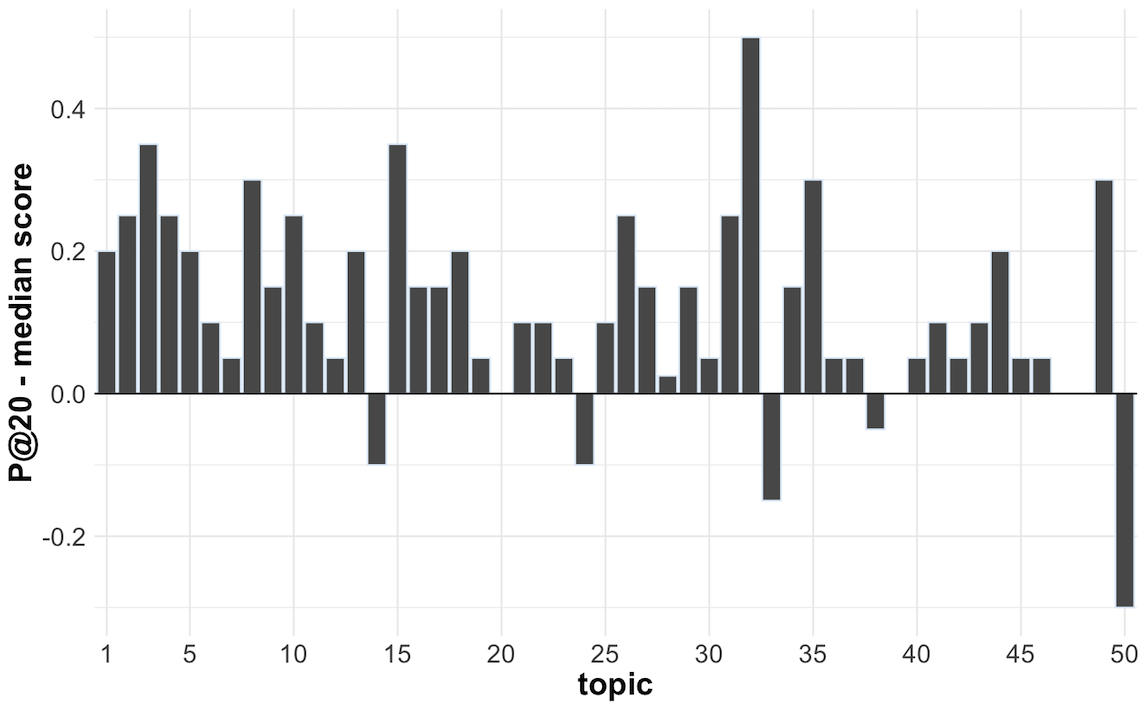
Per topic performance difference between our best model (model 7) and the median of all official submissions for the precision at document 20 (P@20) metric in round 5.

The difference in performance per topic between our best model and the median of the submitted runs in round 5 for all teams for the P@20 metric is shown in Figure 6. Indeed, topics 11, 12, and 19 seemed hard for all the models participating in the TREC-COVID challenge to retrieve the correct documents. Even if our best model had poor performance for those topics, it still outperformed most of the runs submitted to the official evaluation. In particular, topic 19 had only 9 relevant or somewhat relevant documents in the official relevance judgments, which means that its max performance can be at most around 50% for the P@20 metric. For our worst performing topics compared to the other participants — topics 33 and 50 — better tuning between the ranking weights of the bag-of-words, masked language, and logistic regression models could have boosted the results.

### Time-Dependent Relevance Analyses

Given the dynamics of the COVID-19 pandemic, with a relatively well-defined starting period, a particularly effective technique to remove noise from the results, also adopted by some other participating teams [22], is filtering documents based on their publication dates. For our first-stage retrieval models, we filtered out publications before December 2019 when the outbreak was first detected. This led to a small negative impact on recall but highly improved the precision of our models.

**Figure 7:**
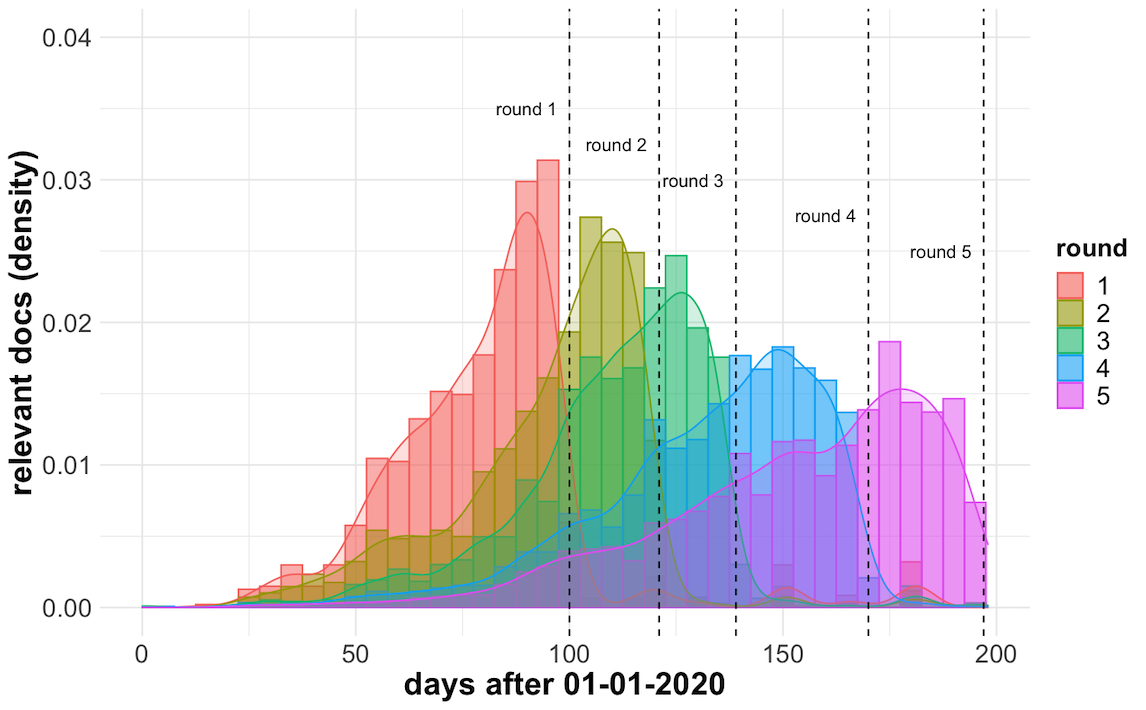
Distribution of the publication dates of the “highly relevant” articles for each of the TREC-COVID rounds.

To better understand how the document relevance varied over time, we analyzed the publication date of the official relevance judgments for the 5 rounds of TREC-COVID. As we can see in Figure 7, there is a clear exponential decay pattern in the number of relevant articles over time for all the rounds, with a faster decay in the first rounds and a longer tail for the later ones. We noticed that more recent publications closer to the round start, when the snapshot of the collection was created and queries were submitted, tended to have a higher probability of being relevant to the information need, with a half-life of around 20 days for round 1. This is somehow expected. First, as the documents found in previous query rounds were explored and are no longer relevant, only the most recent data are interesting, particularly in the gap between rounds. A second explanation is that in the case of a pandemic, new evidence arrives at an explosive rate, possibly refuting older knowledge.

## Discussion

To support effective search and discovery of COVID-19–related relevant literature in the COVID-19 infodemic, we explored the use of a multistage retrieval pipeline supported by bag-of-words models, masked language models, and classic learning-to-rank methods. The proposed methodology was evaluated in the context of the TREC-COVID challenge and achieved competitive results, being ranked in the top 4 of 126 runs among 28 teams participating in the challenge. The use of the multistage retrieval approach significantly improved the search results of COVID-related literature, leading to a gain in performance of 25.9% in terms of the NDCG@20 metric compared to a bag-of-words baseline. Particularly, the ensemble of masked language models brought the highest performance gain to the search pipeline. Indeed, ensembles of language models have proved to be a robust methodology to improve predictive performance [53-55].

The COVID-19 pandemic has led to a huge amount of literature being published in the most diverse sources, including scientific journals, grey repositories, and white reports, among others. As the pandemic continues, the number of scientific publications grows at an unprecedented rate causing an infodemic within many of the different disciplines involved [3]. Finding the most relevant information sources to answer different information needs within the huge volume of data created had become of utmost necessity [2]. By enabling the discovery of relevant information sources to complex user queries, effective retrieval models as proposed in this work may help to tackle the spread of misinformation. Such models empower experts with a minimal cost to search and discover information sources within a massive and fast-evolving corpus. Indeed, our model provides relevant information sources for more than 8 documents in the top-10 rank. Thus, continuous active search methods could be put in place to monitor and discover sources of evidence to certain query topics of relevant public health interest (eg, “coronavirus origin”) in a timely manner. This in turn, would enable experts to analyze, identify, and curate both sources of the best evidence at the time and sources of misinformation. The former would foster the creation among others of living systematic reviews [56,57], which is one of the recommendations of the WHO to tackle the COVID infodemic [2]. On the other hand, the latter could help fight, for example, the spread of scientific fake news by early retraction of misinforming articles, particularly in preprint servers, and thus limiting their exposition.

Looking at the boost in performance of model 3 (mlm + rrf) alone, one could be tempted to argue that masked language models could be the main component in a retrieval system. However, 2 issues may arise: algorithmic complexity and search effectiveness. The former is related to the high complexity of masked language models *(O(n^2^ · h)*, where *n* is the sentence length and *h* is the number of attention heads), which makes it prohibitive to classify a whole collection, often containing millions of documents, for every given query. The latter is related to the effectiveness of the individual models themselves. As shown in Table 5, individually, the performance of the language models is not significantly different from the baseline BM25 model. Thus, we believe it is the combination of models with different properties that can provide a successful search strategy in complex corpora, such as the one that originated from the COVID-19 infodemic.

In terms of practical implications, by effectively processing natural language, the methodology proposed can help biomedical researchers and clinicians to find the COVID-19 papers that they need. The efficient literature discovery process fostered by our methods may lead to faster publication cycles when required, for example reducing from weeks to days the drafting time of COVID-19 reviews [58], but also to less costly creation of curated living evidence portals, which will inform clinicians and public health officers with the best available evidence [59]. Indeed, as shown in [27,60], these methodologies outperform commercially available tools for searching and discovering COVID-19–related literature. Moreover, as they are data-driven, it is expected that they can be extrapolated to other types of corpora, such as clinical trial protocols and biomedical metadata datasets [60,61], enabling thus a more comprehensive identification of scientific evidence. Equally important, as the COVID-19 infodemic is not the first and unlikely the last [62,63], our methodology and findings could be extended to help tackling future epi-, pan-, and infodemics by supporting relevant actors to scan large and fast-changing collections to create timely reviews and curated evidence and apply localized infodemic management approaches.

With the rapid surge of published information and the variety of topics and sources related to COVID-19, it became hard for professionals dealing with the pandemic to find the correct information for their needs. While the automation discussed in this work can support more effective search and discovery, some high-level topics are still challenging. Indeed, some topics assessed in the TREC-COVID challenge were shown to be particularly hard for the retrieval models. For example, for topic 11, which searched for documents providing information on “guidelines for triaging patients infected with coronavirus,” our best model prioritized documents providing information about indicators for diagnosis (eg, “early recognition of coronavirus,” “RT-PCR testing of SARS-CoV-2 for hospitalized patients clinically diagnosed”). On the other hand, it missed documents including passages such as “telephone triage of patients with respiratory complaints.” Similarly, for topic 12, which searched information about the “best practices in hospitals and at home in maintaining quarantine,” our model prioritized documents providing information about “hospital preparedness” (eg, “improving preparedness for,” “preparedness among hospitals”) and missed documents containing information about “home-based exercise note in Covid-19 quarantine situation.”

The methodology proposed has some limitations. First, it fails to explore transfer learning of learning-to-rank datasets. While the top-ranked teams all used multistage retrieval approaches, confirming the value of such methodology in modern retrieval models [18,23], the reranking strategy within the different pipelines varied slightly among the participants. For example, the top-ranked team used transfer learning from the MS MARCO learning-to-rank dataset and from a zero-shot learning approach. Other teams in the top 3 used transfer learning from a silver collection, based on the known item search technique [64]. Second, while we explored the combination of different topic items to build our queries, we failed to work on the document indexing unit, leaving all the normalization work to the probabilistic weighting models. As the COVID-19 literature comes from heterogeneous collections, containing sometimes only title and sometimes large full text, even with good finetuning of the model parameters, such variation in size and content poses a challenge to the first-stage retrieval model. Indeed, some strategies that explored decomposing the indexing unit into small structures, such as sentences and paragraphs, have achieved more competitive results [23].

Another limitation of our work was the ability to explore the freshness of the corpus. The TREC-COVID challenge dynamics, running throughout a sequence of rounds with new incremental search topics added on each round, provides an interesting setting for evaluating retrieval models in an infodemic context. It simulates typical search and discovery workflows, in which evolving queries are posed against an evolving body of knowledge over time, and already discovered documents in previous searches are no longer relevant [65,66]. A successful strategy in this case is to filter out results according to a cut-off date, thus reducing noise in the retrieval set. However, in retrospect, we noticed that another useful technique, which is very natural to an infodemic case, could be to decay the score of publications by their distance to the present time or explore their recency or freshness [67,68], as highlighted in Figure 7, rather than a hard cut-off (ie, December 2019 in our case) for all the rounds. We leave exploring such a strategy as future work.

To conclude, we believe our information retrieval pipeline can provide a potential solution to help researchers, decision makers, and medical doctors, among others, search and find the correct information in the unique situation caused by the COVID-19 pandemic. We detailed the different components of this pipeline, including the traditional index-based information retrieval methods and the modern NLP-based neural network models, as well as insights and practical recipes to increase the quality of information retrieval of scientific publications targeted to the case of an infodemic. We grounded our results in the TREC-COVID challenge, where around 50 different teams participated in the 5 rounds of the competition. We showed very competitive results as judged by the official leaderboard of the challenge. Apart from the COVID-19 case, we believe our solutions can also be useful for other potential future infodemics.

## Abbreviations

BERT: Bidirectional Encoder Representations from Transformers
BM25: Okapi Best Match 25
Bpref: binary preference
CORD-19: COVID-19 Open Research Dataset
DFR: divergence from randomness
LMD: language model Dirichlet
MAP: mean average precision
MERS: Middle East respiratory syndrome
MS MARCO: Microsoft Machine Reading Comprehension
NDCG: normalized discounted cumulative gain
NLP: natural language processing
PMC: PubMed Central
RoBERTa: robustly optimized BERT approach
RRF: reciprocal rank fusion
SARS: severe acute respiratory syndrome
tf-idf: term frequency-inverse document frequency
WHO: World Health Organization
